# Group‐based pelvic floor muscle training for pregnant women: A randomized controlled feasibility study

**DOI:** 10.1111/jan.16365

**Published:** 2024-08-14

**Authors:** Xiaowei Yang, Lynn Sayer, Sam Bassett, Sue Woodward

**Affiliations:** ^1^ Florence Nightingale Faculty of Nursing, Midwifery and Palliative Care King's College London London UK; ^2^ Department of Clinical Teaching and Research Nanjing Vocational Health College Nanjing China

**Keywords:** group‐based intervention, mixed methods, nursing, nursing intervention, pelvic floor muscle training, pregnant women, process evaluation

## Abstract

**Aim:**

This study aims to test the feasibility and acceptability of a group‐based pelvic floor muscle training for pregnant women in China and facilitate women's adherence to the pelvic floor muscle training programme.

**Background:**

Urinary incontinence is a prevalent health problem in women worldwide, especially in pregnant women. Supervised pelvic floor muscle training is recommended as the first‐line conservative treatment for urinary incontinence. However, the implementation and effectiveness of pelvic floor muscle training are limited by insufficient human resources and low adherence. Group‐based interventions may improve people's adherence to interventions by facilitating peer support. However, it has been investigated in a limited number of maternity studies.

**Design:**

Feasibility testing randomized controlled trial, accompanied by a mixed methods process evaluation.

**Methods:**

This study was guided by the Medical Research Council framework for complex interventions and the Behaviour Change Wheel guide to developing interventions. A three‐phase, mixed‐methods design was used in this study. This study reported the feasibility of the group‐based pelvic floor muscle training programme. Semi‐structured reviews were conducted following the intervention to explore the acceptability of the programme.

**Results:**

The study included 48 pregnant women with a recruitment rate of 52.17%. The adherence rate to the training programme was 66.67%. The intervention was positively valued, in particular the support promoting participants' adherence, but additional changes need to be made to the programme for a future trial.

**Conclusions:**

Group‐based pelvic floor muscle training programme provides a possible way of delivering pelvic floor muscle training with limited health professionals in China. The study showed promising results concerning the acceptability and feasibility of the intervention, which were well perceived by both pregnant women and the midwife.

**Implications for Patient Care:**

Group‐based pelvic floor muscle training may have the potential of reducing the prevalence of urinary incontinence in pregnant women with insufficient healthcare professionals.

**Impact:**

- This study assessed the feasibility of delivering group‐based pelvic floor muscle training in pregnant women in China.
- The group‐based pelvic floor muscle training is acceptable to both pregnant women and the midwife, but integrating online and face‐to‐face sessions need to be considered.
- The findings of this study provided evidence for delivering group‐based pelvic floor muscle training to pregnant women in China.

**Reporting Method:**

The study has adhered to CONSORT guidelines (Table S1) and TIDier checklist (Table S2).

**Patient and Public Contribution:**

The patient and public have been invited as stakeholders during the development of the intervention. They worked with healthcare professionals to co‐design the group‐based pelvic floor muscle training programme.

**Registration:**

The trial was registered on ClinicalTrials.gov (NCT05242809) under the title ‘Development and Feasibility Testing of a Group‐based PFMT Programme for Antenatal Women in Nanjing City in China’.

**What this paper adds**

- It is feasible to implement group‐based pelvic floor muscle training in pregnant women in China.
- The group‐based pelvic floor muscle training is acceptable to both pregnant women and the midwife, but integrating online and face‐to‐face sessions need to be considered.
- Group‐based pelvic floor muscle training showed potential effectiveness in improving pregnant women's urinary incontinence symptoms.

## 1 INTRODUCTION

Urinary incontinence (UI) is a common health condition which is defined as involuntary loss of urine (D'Ancona et al., 2019). Millions of women suffer from this problem worldwide. Population‐based research has shown that urinary incontinence affects roughly 30% (ranging from 20.5% to 68.8%) of adult women (Zengin, 2010). A systematic review which aimed to assess the prevalence and incidence of UI in pregnant women in the Western world showed that an overall mean prevalence of UI during pregnancy is 41% with a range of 9%–75% (Moossdorff‐Steinhauser et al., 2021). While the prevalence of UI in pregnant women in China varies from 7.73% (Huang et al., 2016) to 36.00% (Wang, 2009). Treatment options range from altering one's lifestyle to intrusive surgical procedures (NICE, 2019). Women who are otherwise healthy may prioritize treating their urine incontinence by actively participating in pelvic floor therapy, lifestyle modifications (such as optimizing fluid intake), medication or surgery to manage chronic problems (Aoki et al., 2017). The clinical practice guidelines recommended individualized pelvic floor muscle training (PFMT) as a first‐line conservative treatment for stress urine incontinence and any type of UI (NICE, 2019). However, the lack of appropriately qualified health professionals prevents PFMT from being implemented in many nations (Bland et al., 2003). There is a need to look into ways to implement PFMT into clinical practice at scale in order to reach more women and get over the human resource barriers. Therefore, this study aimed to assess the feasibility and acceptability of a group‐based PFMT programme for pregnant women in Nanjing, China.

## 2 BACKGROUND

UI is a highly widespread disease that has a significant impact on health and quality of life (Biswas et al., 2017). The most common cause of incontinence in women is dysfunction of the pelvic floor muscles or bladder, which frequently occurs during pregnancy, childbirth or menopause (Aoki et al., 2017). Some women with the symptoms of UI experience enough inconvenience and intrusion from urine incontinence to warrant considering treatment. PFMT was not implemented widely although it has been recommended to be the first‐line treatment for UI. Group‐based PFMT provides an efficient way to prevent and treat UI (Dumoulin et al., 2020). It is reported that compared to individualized training, group‐based PFMT provided at least 60% in cost savings and sustained a persistent decrease in UI episodes over 1 year in older women (Brazier et al., 2008; Cacciari et al., 2022). In addition, the high adherence to the training sessions and low drop‐out rate of the group‐based pelvic floor muscle training in older women indicated the acceptability of the group‐based PFMT (Frechette‐Chaine et al., 2018).

In China, PFMT provision is varied. Although the basic examination to assure the safety of the pregnant women and the foetus is required by the government and provided equally, the information on PFMT provided by different hospitals is not the same even in the same city because both antenatal and postnatal PFMT is not included in routine care. Compared to women after delivery who have the opportunity to receive supervised PFMT and paid for privately, pregnant women were only able to receive verbal information on PFMT in these hospitals. Although sometimes pregnant women do not experience UI in their first or second trimester, it is important to deliver supervised PFMT to them for prevention, because it was reported that the prevalence of UI rises with the gestational period from 9% in the first trimester to 34% in the third trimester (Moossdorff‐Steinhauser et al., 2021). In addition, a Cochrane review found that providing antenatal PFMT to women reduces UI risk in their late pregnancy. It is also in line with cost‐effectiveness analysis which supports offering PFMT to all pregnant women for UI prevention, emphasizing group sessions over individual postnatal treatments if at least four women can attend (Brennen et al., 2021).

This study was a feasibility study which was guided by the Medical Research Council framework for the development of complex interventions (Skivington et al., 2021). Following the framework, this study was conducted to test the feasibility and acceptability of the group‐based PFMT programme. This study also included a nested qualitative interview to evaluate the perceptions of the intervention from pregnant women with or without UI and the midwife who delivered the training sessions. In addition, this study provided information on necessary changes to the protocol of the group‐based PFMT programme to increase its acceptability in a full‐scale effectiveness trial.

## 3 METHODS

### 3.1 Aims and research design

A single‐centre, two‐arm feasibility randomized controlled trial (RCT) of the group‐based PFMT was conducted with pregnant women in Nanjing City in China between July 2022 and January 2023. This study aimed to assess the feasibility of delivering group‐based PFMT to pregnant women in China. The recruitment rate, retention rate and acceptance of the group‐based pPFMT programme were measured.

### 3.2 Setting and participants

The study site was an obstetrics and gynaecology hospital in Nanjing City with approximately 26,000 babies born in this hospital every year. Participants were included if they volunteered to be part of the study, were nulliparous women who were aged 18 years and older, have singleton foetus and the gestational age was 19–24 weeks with or without the symptom of UI, also, they were capable of giving valid informed consent. Participants were excluded if they had a pre‐existing UI symptom before pregnancy or had pregnancy complications, urinary tract infection, history of urogenital surgery or diseases or were at high risk of preterm labour. The details of eligibility criteria for participants have been reported in the protocol.

### 3.3 Sample and procedure

The target sample size for this study was 48 participants, 24 in each arm. The reason for why including 48 participants has been explained in the protocol of this study (Yang et al., 2023). The pregnant women who were referred to Nanjing Maternity and Child Health Care Hospital at 19–20 gestational weeks were provided with the study information by the midwives. They were screened for eligibility if they volunteered to participate in the study. An information sheet was provided to the eligible participants and the participants were telephoned by the principal researcher to address any additional questions. The principal researcher then arranged a time for the participants to sign consent forms after verifying their understanding of what the study involved and confirming their interest in participation. Screening and recruitment stopped when the target sample size of 48 with 24 in each arm was reached.

### 3.4 Blinding

It was not possible for the research midwife to be blinded. Also, the principal researcher was not blinded to the participants when conducting interviews with the pregnant women and the midwife after the intervention. However, the principal researcher was blinded to the allocation when doing the baseline and follow‐up data analysis.

### 3.5 Intervention group (the group‐based pelvic floor muscle training)

Participants in this group received four face‐to‐face sessions which were delivered by the research midwife in Nanjing Maternity and Child Health Care Hospital. There were three groups with eight participants in one group. The sessions were delivered once a month to correspond to the time of regular antenatal check (around 24, 28, 32 and 36 gestational weeks). Participants were also encouraged to do PFMT twice a day at home. They were provided with (i) training booklets which contained information on PFMT, (ii) a training diary which recorded the training undertaken and symptoms of UI and (iii) stickers which could be pasted anywhere to remind them to do exercises. In addition, they were invited to enter a WeChat group in which they could discuss and share any information or experience on their PFMT. Reminders and audio which were sent automatically twice a day in the WeChat group to guide the pregnant women to do PFMT.

### 3.6 Control group (usual antenatal care)

The participants in the control group received usual antenatal care. The doctors recommend pregnant women attend antenatal classes which include information on PFMT and were run by midwives, but no further information or support materials were provided to the pregnant women.

### 3.7 Feasibility outcomes and outcome measures

It was possible to assess the practicality of recruiting participants and willingness to be randomly assigned by calculating the percentage of participants invited to take part in the study who were eligible, participants who chose to take part in the study and the reasons for ineligibility and participation refusal. Adherence to the programme was evaluated by the attendance of face‐to‐face sessions and completion of a training diary. The frequency of undertaking PFMT at home, completion rates of the baseline data and outcome measures at 37 gestational weeks and 42 days after delivery were also recorded. The progression criteria were published in the protocol of this study (Yang et al., 2023).

The age, education status, body mass index, gestational weeks and the International Consultation on Incontinence Questionnaire–Urinary Incontinence Short Form questionnaire (ICIQ UI‐SF) were collected at baseline, around 37 gestational weeks and 42 days after delivery. The symptom and the impact of UI on daily life was self‐reported by using the questionnaire. Attendance registration was recorded by the midwife and training diaries were checked by the midwife at the beginning of each session. The registration and training diaries were monitored to track adherence to the programme. The detail of when and how these measurements were collected was presented in the protocol (Yang et al., 2023).

The feasibility of delivering the group‐based PFMT programme was assessed through semi‐structured interviews following the completion of the intervention. Six pregnant women in the intervention group were purposively sampled from the intervention group to attend semi‐structured interviews in groups with the principal researcher. Then a semi‐structured interview was conducted by the principal researcher with the research midwife. The interviews were digitally audio‐recorded, anonymized and transcribed verbatim by the principal researcher. The interviews were conducted face‐to‐face and analysed by the principal researcher.

### 3.8 Process evaluation

A process evaluation which aimed to gain information on the feasibility and the acceptability of the programme was conducted in this study. The logic model was used to monitor intervention fidelity and provide insight into how the intervention did or did not work in practice, identify any unintended consequences and refine the design of the future trial. The logic model used in this study was discussed with the stakeholders (published in the protocol (Yang et al., 2023)) and was refined after the conduction of the intervention.

### 3.9 Data analysis

SPSS 26.0 was used for data analysis in this study. The dichotomous feasibility outcomes were presented by number and proportion. Any withdrawals from the intervention were recorded by the midwife. Completion rates of the baseline and follow‐up outcome measures at 37 gestational weeks and 42 days after delivery were recorded.

Descriptive frequency analysis was used for baseline characteristics. For categorical variables, the frequencies and percentages were calculated. Descriptive analyses of participants' characteristics were expressed as a mean (standard deviation). The Wilcoxon test and paired t‐test were used for comparisons between baseline and follow‐up in the intervention and control group, respectively. All tests were two‐sided and *p* < .05 was considered statistically significant. To measure the differences in perceived impact score and ICIQ UI‐SF score between the control group and intervention group, the effect size and the 95% confidence interval (CI) were calculated.

The interviews were transcribed and coded using qualitative thematic analysis (Braun & Clarke, 2006) following the six phases of thematic analysis.

### 3.10 Ethical considerations

The trial was registered before the commencement of recruitment (NCT05242809) and institutional ethics approval was obtained from King's College London Research Ethics Committee (LRS/DP‐21/22–26,714) and Nanjing Maternity and Child Health Care Hospital (2021KY‐084). The protocol of this study was published elsewhere (Yang et al., 2023).

## 4 RESULTS

### 4.1 Characteristics of the participants at baseline

The recruitment started in August 2022 and the follow‐up data was collected until February 2023. The participants were aged from 24 to 36 years and the majority had received higher education (47/48, 97.92%). The body mass index of the participants ranged from 19.33 to 25.53. There were 14 out of 48 pregnant women reporting symptoms of UI with seven in the intervention group and seven in the control group. Two of the pregnant women reported urine leaks before they get to the toilet and the other 12 pregnant women with UI all leak urine when they cough or sneeze or leak when they are physically active/exercising. Among the 14 pregnant women with UI, three pregnant women leaked urine more than once a week. No significant differences were found between the intervention group and the control group at baseline (Table 1).

**TABLE 1:** Characteristics of the participants and baseline measurements.

Characteristic	Intervention group (*n* = 24)	Control group (*n* = 24)	*Z*/*t*	*p* value
Age, year				
24–30	18 (75)	14 (58.33)		
31–35	6 (25)	9 (37.5)		
Above 35	0 (0)	1 (4.17)	1.281	.2
Education^a^				
Primary school or below	0 (0)	0 (0)		
Junior or senior school	0 (0)	1 (4.17)		
University	20 (83.33)	18 (75)		
Postgraduate or above	4 (16.67)	5 (20.83)	−0.058	.953
Body mass index^c^, mean ± SD, kg/m^2^	22.16 ± 2.02	21.78 ± 2.91	−0.581	.565
Gestational weeks, Mean ± SD	19.4 ± 0.89	19.33 ± 0.58	0.859	.393
ICIQ UI‐SF				
Frequency of incontinence episodes^b^				
Never	17	17		
About once a week or less often	7	4		
Two or three times a week	0	1		
About once a day	0	1		
Several times a day	0	0		
All the time	0	1	−0.272	.785
Amount of leakage				
None	17	17		
Small	6	5		
Moderate	1	2		
Severe	0	0	−0.091	.928
Perceived impact of those reporting UI	0.88 ± 1.65	1.17 ± 1.83	0.579	.565
ICIQ UI‐SF score	1.83 ± 3.10	2.67 ± 4.04	0.802	.427

^a^ Values are presented as *n* (%), Wilcoxon sign‐rank test (*Z*) and paired *t*‐test (*t*).

^b^ Comparison in age, education, frequency of incontinence episodes and amount of leakage between the two groups used the Wilcoxon sign‐rank test.

^c^ Comparison in body mass index, gestational weeks, perceived impact score and ICIQ UI‐SF score between the two groups used paired *t*‐test.

### 4.2 Feasibility outcomes

**FIGURE 1:**
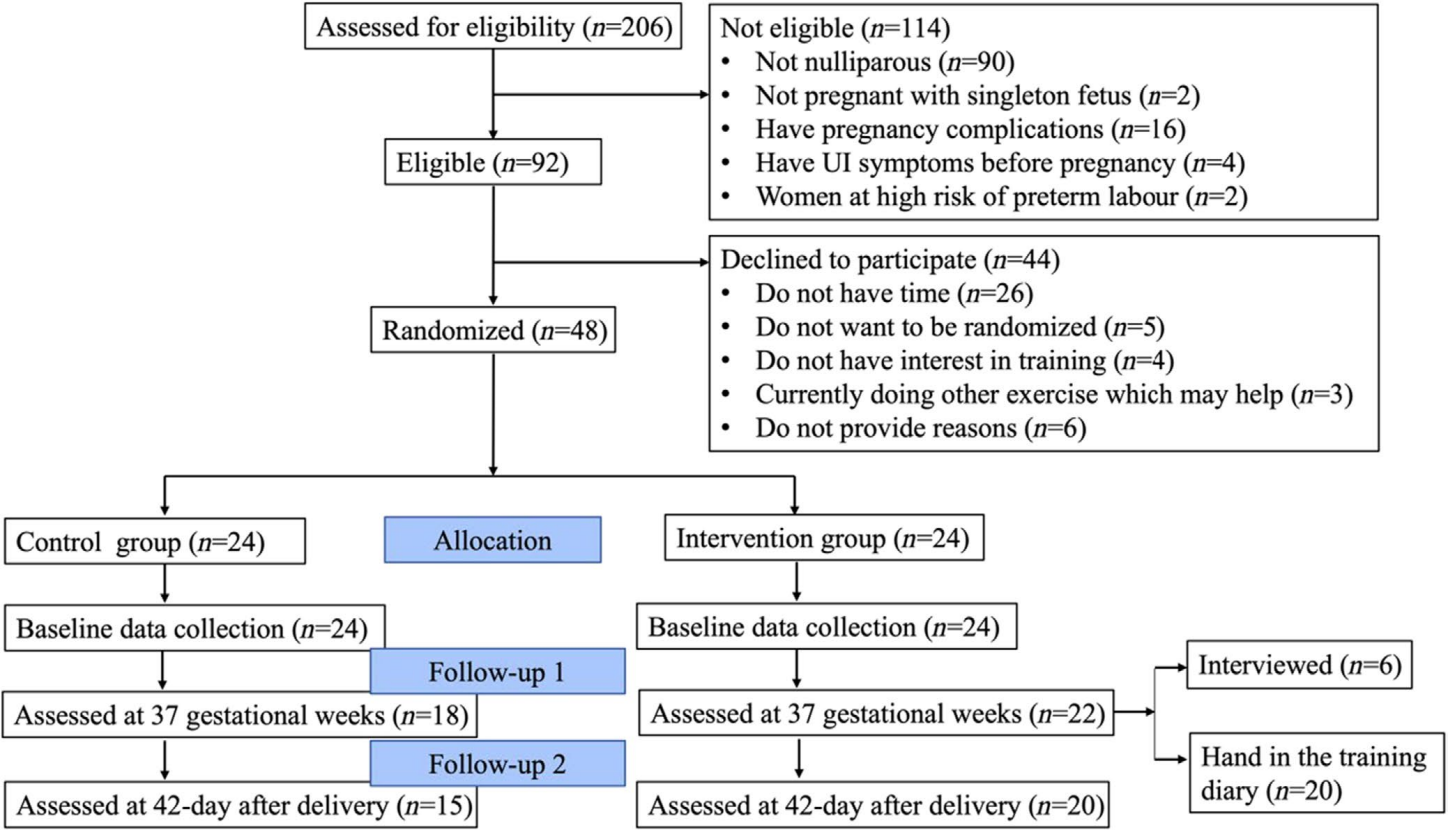
Consort flow diagram.

A total of 206 women were referred to Nanjing Maternity and Child Health Care Hospital and 92 of them were eligible for the study. A total of 48 eligible participants consent to take part in the programme with a recruitment rate of 52.17%. Of the pregnant women who declined to participate, 59% (26/44) provided the reason for the time commitment required for the study. The other reasons included not wanting to be randomized, not interested in training and currently doing other exercises that they think may help. The study flow chart is presented in Figure 1.

### 4.3 Completeness of outcome data measures, compliance with the training and retention

For baseline data collection, the completion rate was 100% both in the intervention group and the control group; however, data collection at 37 gestational weeks received 18 questionnaires (75%) in the control group and 22 questionnaires (91.67%) in the intervention group. The follow‐up data collection at 42 days after delivery received 15 questionnaires (62.5%) in the control group and 20 questionnaires (83.33%) in the intervention group. A total of 20 pregnant women in the intervention group handed in the training diary (1). The training diary indicated almost all women did pelvic floor muscle training twice a day; however, there were two pregnant women who did not record the frequency of undertaking PFMT from the second training session of the programme onwards.

All 24 participants who consented and were randomized to the intervention group started the intervention. Of 24 pregnant women in the intervention group, 29.17% (7/24) of the women attended all the sessions and 66.67% (16/24) participants attended at least three sessions. All these women are considered to be adherent to the programme. For the participants who did not attend the sessions, the main reason given was that they did not have time to attend the sessions while waiting for the other physical examinations.

In summary, the recruitment rate met the progression criteria to proceed with the randomized controlled trial, while retention rate and adherence to the training programme partly met the progression criteria and some measures met the progression criteria to proceed with changes (Table S3).

### 4.4 Initial estimates of potential efficacy outcome measures

The ICIQ UI‐SF was sent to the participants at around 37 gestational weeks and 42 days after delivery. The means and standard deviations for outcome measures are presented in Table 2. Compared to the control group, the group‐based PFMT at 37 gestational weeks only showed a possible protective effect in reducing perceived impact score [mean difference (MD) 0.929; 95% confidence interval (CI) −0.284–2.142; *t* = 1.551, *p* = .129] and ICIQ UI‐SF score [MD 2.222; 95% CI −0.391–4.836; *t* = 1.721, *p* = .093]. However, there was a significant reduction in both perceived impact score [MD 1.383; 95% CI 0.113–2.654; *t* = 2.215, *p* = *.034*] and ICIQ UI‐SF score [MD 3.267; 95% CI 0.721–5.813; *t* = 2.610, *p* = *.013*] at 42 days after delivery in the intervention group (Table 2).

**TABLE 2:** Means and standard deviations of ICIQ‐SF score at 37 gestational weeks and 42 days after delivery.

ICIQ UI‐SF	37 gestational weeks		*Z*/*t* value	*p* value	42 days after delivery		*Z*/*t* value^a^	*p* value
	Intervention group (*n* = 22)	Control group (*n* = 18)			Intervention group (*n* = 20)	Control group (*n* = 15)		
Frequency of incontinence episodes fub								
Never	14	8	−1.406	.219	13	5	−2.161	.055
About once a week or less often	7	7			7	7		
Two or three times a week	1	1			0	2		
About once a day	0	1			0	1		
Several times a day	0	0			0	0		
All the time	0	1			0	0		
Amount of leakage^b^								
None	14	8	−1.452	.199	13	5	−1.851	.092
Small	6	5			7	5		
Moderate	2	5			0	5		
Severe	0	0			0	0		
Perceived impact of those reporting UI^c^	1.44 ± 1.89	2.11 ± 1.99	1.551	.129	1.53 ± 1.88	2.53 ± 1.92	2.215	**.034**
ICIQ UI‐SF score^c^	3.06 ± 3.70	4.72 ± 4.62	1.721	.093	2.93 ± 3.35	5.47 ± 4.26	2.610	**.013**

^a^ Wilcoxon sign‐rank test (*Z*), paired *t*‐test (*t*).

^b^ Comparison in the frequency of incontinence episodes and amount of leakage between the two groups used the Wilcoxon sign‐rank test.

^c^ Comparison in perceived impact score and ICIQ UI‐SF score between the two groups used paired *t*‐test.

Bold indicates significant value <.05

### 4.5 Acceptability and feasibility of the intervention

Of the 24 pregnant women who were randomized to the intervention group, six women were interviewed. In this group of women, one of them had symptoms of UI and five of the women attended at least two sessions. Following the interview with the participants, the midwife's experience of delivering the group‐based PFMT programme and her opinions on the acceptability of delivering the intervention and suggestions for future implementation were also explored by using a semi‐structured interview. The feedback from the participants was discussed with the midwife in the interview. Three main themes that emerged from the interviews and a few sub‐themes emerged and were linked to the main themes.

#### 4.5.1 Acceptability of the programme

The following quotes have been translated by the principal researcher.

##### Supporting and encouraging each other

The participants mentioned that the group format was supportive because they could get peer support from each other. Personal information of the participants had not been disclosed and they were allowed to be anonymous in the group which made them feel that their information was protected. They could share their experience in doing exercises and felt encouraged by other participants' active engagement.

Compared to individualized pelvic floor muscle training, I preferred group because I did not feel isolated and had the passion to share my experience. I got support from the group members(Participant 3)

I did not feel embarrassed to share my experience because the group members did not know each other before. (Participant 2)

##### Positive views on the regimen and supporting materials

The participants thought the number of contractions, the frequency of exercising and the time spent exercising were acceptable. They reported that it was also acceptable for women to be randomized either to the intervention group or the control group. However, the interview was performed with the participants in the intervention group only.

The group intervention not only taught the pregnant women in groups and created a WeChat group for the pregnant women to communicate but also provided other support, for example, providing the PFMT booklet and training diary, stickers and sending reminders and audio instruction in the WeChat group. The participants thought the reminder and the audio instruction sent following the reminder were useful in helping them perform the training consistently. Also, the provided stickers were mentioned as being helpful. They could paste the sticker anywhere to remind themselves to do the training (e.g., the edge of the office computer screen or on the mirror in the bathroom). Also, in the midwife's opinion, the provided training package was considered a good resource in supporting the delivery of the programme and facilitating participants' behaviour change. The participants valued integrating exercises into everyday life. They thought integrating the PFMT with the time before daytime naps and sleep helped to build a habit of exercising, which may enable them to remember to do pelvic floor muscle training in the long term.

The overall exercise plan is good, and the arrangement is acceptable. The two times of exercise scheduled before the nap and sleep were quite reasonable. I appreciate the audio sent in the group, so I could practice consistently along with the audio. (Participant 1)

#### 4.5.2 Issues need to be considered for future study

##### Fees for the supervision sessions

Although the participants received training supervision sessions free of charge in this study, one participant asked about how much the training sessions would cost for future training sessions. A discussion on the acceptable fees was carried out between the principal researcher and the pregnant women. The pregnant women suggested that the fees charged by the hospital should not be much higher than private rehabilitation institutions because compared to the individualized training, the participants thought they would receive less attention in group training and lower fees charged by the hospital would attract more pregnant women to participate in the programme.

I think the fees should not be much more than that of private institutions, since they generally do not require an appointment in advance, and I can receive pelvic floor muscle training immediately upon arrival. Although their service team is undoubtedly not as professional as midwives in the hospital, the services offered by them are somehow convenient…… I prefer receiving training in the hospital if the fees charged by the hospital and private institutions are the same…… Of course, I think more pregnant women would like to receive pelvic floor muscle training supervision if the hospital charges even less than the private institutions. (Participant 2)

The fees for current postpartum pelvic floor muscle training in the hospital are acceptable, but that is individualized service. I think the group‐based training should charge fewer than individualized sessions. (Participant 6)

##### Workload

Rather than one‐on‐one training supervision, delivering PFMT in groups was considered a cost‐effective approach. However, the workload of the health professionals may be a barrier to delivering the programme to more women. The four sessions (each lasting for 45 min) delivered in the training were recognized as a significant amount of time if the groups of participants increased. Midwives and nurses in the hospital would not have enough time to deliver the intervention and if they made the time, the other services provided by them would suffer. Although the midwife believed that she and the medical team would benefit from adequate training on delivering PFMT, she did not think she and her colleagues could manage to deliver the interventions if the groups of participants increased. It was thought that an additional midwife would be needed in a full‐scale study.

I did learn a lot from delivering the training, but I think it is a lot of work to deliver four sessions for each group of women. The current number of midwives in the hospital was not enough if the number of groups increased. It is necessary to have an additional midwife to manage the whole process of the programme. (The midwife)

##### Time allocation for the face‐to‐face sessions

All the participants in the interview mentioned the timing of the face‐to‐face sessions. Although the sessions had been planned to coincide with regular antenatal visits, not having time to attend sessions was a difficulty that was repeatedly mentioned. Although they knew the importance of doing PFMT and receiving supervision from health professionals, attending regular antenatal checks was prioritized because the women needed to get all the results of the physical check before booking the next appointments with the doctors. Whereas attending the face‐to‐face PFMT supervision was not compulsory for booking the next appointment. Pregnant women may need to stay in the hospital for a long time if they cannot get the results of the physical examination within a half day. Therefore, some of the participants gave up and some of the participants were late for the sessions because they did not want to lose their place in a queue, thus keeping other women in the same training group waiting. The midwife agreed that she had to wait for the late participants to ensure the training was delivered in planned groups resulting in the training sessions starting up to 12 min late. Sometimes participants joined the sessions after the midwife had started. Progression criteria need to be provided in advance to decide how long is it acceptable to wait before starting, whether participants can join after the session has started and how many absent women in one group is acceptable for a session to start.

When asked about suggestions to improve the attendance rate of face‐to‐face sessions, the participants suggested making some of the sessions online but retaining the skill training face‐to‐face so that they can be motivated by the midwife to do the exercise and be corrected in time if they performed in the wrong way. Running loop sessions could be another choice, then the participants can choose the time of attending sessions themselves. The midwife also suggested delivering a part of the training sessions online to increase the attendance rate which was consistent with the participants' suggestions. For running loop sessions which provide a flexible time for participants, the midwife suggested that the workload to provide loop sessions needed to be taken into consideration. Another possible way was to develop a booking system for pregnant women who would like to attend the sessions to automatically adjust the time of other antenatal checks.

My maternity leave only lasts for a half day. To schedule an appointment with the doctor for the next antenatal check, I need to complete all of the physical checks within the half day. Sometimes, I only have time to complete the physical checks. (Participant 3)

I believe that face‐to‐face sessions can more effectively motivate us to keep doing pelvic floor muscle exercises, although online sessions are more convenient. Perhaps you can consider providing loop sessions so that we can attend the sessions whenever we have time. If you did, perhaps a few more people would attend the sessions than there are today. (Participant 4)

Many pregnant women were late for the sessions or did not attend the sessions without asking for leave in advance. I needed to call them to confirm whether they were late or not attending the session. It was a waste of time. The limit on time for waiting need to be set in advance. (The midwife)

#### 4.5.3 Need for refinement

##### Ground rules need to be emphasized

The participants mentioned sometimes it was difficult for them to identify the reminder and audio instruction sent in the WeChat group because of the volume of discussion posted by the participants leading to key information sometimes being overlooked. The ground rules needed to be emphasized to ensure reminders do not get overtaken by discussion between group members.

Sometimes group members discussed some topics that were not related to pelvic floor muscle training and pregnancy. I had to go through a lot of information before I was able to find the key information. (Participant 2)

##### Refinement of the sessions and outcome measures

Participants were taught about the anatomy of pelvic floor muscles, the effectiveness of PFMT and guidance on how to do PFMT correctly in the face‐to‐face sessions. They mentioned that they had learnt a lot about why and how to perform the correct contractions. They reported having heard about PFMT before but discovered through the sessions that they had not been doing exercise in the correct way before the training programme. The content was said to be easy to follow and the content in each session had a good flow. However, the participants suggested simplifying the content taught in the first 15 min, for example, providing less information on the international guidelines and how the pelvic floor muscle works to improve the symptoms of UI which was also a view agreed by the midwife.

Regarding the outcome measures, the questionnaire was acceptable because it was easy to complete. However, the participants suggested reducing the amount of information that needs to be written in the training diary (whether the participants performed the exercises, the number of contractions and the symptoms of UI). The participants suggested retaining the confirmation of exercise and the symptoms of UI and transferring the training diary into an online form which can be easily filled out after the training. Based on the suggestions from the participants, the midwife suggested turning the training diary into an online form which can be filled with the option ‘yes’ or ‘no’. In addition, it was suggested by the midwife to add interactive animation onto the training slides to increase the participants' interest.

The content is quite good for me to understand why the pelvic floor muscle training should be performed. I learned how to do this exercise from the Internet before this training programme, but I think I have not performed it in the right way until I attended this training programme. (Participant 1)

The questionnaire is easy to complete, but filling in the training diary is quite cumbersome. Sometimes I forgot to record it if the training diary was not available at hand when I was doing the training. It would be great if there is an online form that can be recorded at any time. (Participant 4)

You can try to add more animations in the slides, and decrease the number of texts… yes, I agree, there were a lot of the content to be completed in the training diary. You can consider providing yes or no options in an on‐line form to simplify the process of data collection. (The midwife)

#### 4.5.4 Reasons for not following the regimen

The participants mentioned that sometimes they felt tired from work and thought the effectiveness of the training would not be affected by skipping some sets of exercises. Two of the participants thought it was boring to do exercise every day.

I know that pelvic floor muscle training can help me prevent urinary incontinence. But it is hard for me to do it every day. I do not know the reason. It is really boring to do exercise every day. If I did not achieve enough numbers of required contractions in one day and did not do the exercise on the other day, will it have an impact on the results? I believe it may be challenging for you to answer this question. It is really hard for me to do the same thing every day. (Participant 6)

Sometimes I really feel tired. You know, the work I have undertaken was not reduced because of my pregnancy. I sometimes fell asleep as soon as I touched the pillow. I also do not want to make up the missing exercises on the same day because I do not think the two or three missing exercises would have any impact on the effectiveness of training. (Participant 2)

#### 4.5.5 Outcomes of the intervention

The feedback on the outcome of the intervention was generally positive. The participant with the symptom of UI reported the amount and frequency of urine leakage decreased, but this was not confirmed with objective outcome measures such as vaginal examination. The other participants with no symptoms of UI reported a positive behaviour change in doing the exercise.

I feel that both the frequency of urine leakage and the amount of leaked urine decreased. But I still have the symptom of urinary incontinence when coughing and laughing. (Participant 4)

I feel happy because at least I can keep doing a kind of exercise for three months, which did not happen before. (Participant 5)

## 5 DISCUSSION

This study aimed to assess the feasibility of delivering a group‐based PFMT programme to pregnant women in China. Both quantitative and qualitative methods were used to evaluate whether the progression requirements for moving forward to a full‐scale randomized controlled trial were met. This research suggests that group‐based PFMT may be an acceptable approach for pregnant women to prevent and/or treat UI in China, but amendments to the programme are needed to improve the experience of attending and delivering the group‐based PFMT programme.

### 5.1 Feasibility outcomes

The recruitment rate (52.17%) was slightly higher than expected (50%) (according to the progression criteria), but the time commitment required for attending the group‐based PFMT programme was the main reason for pregnant women not taking part, indicating that this factor might be a potential barrier to the recruitment of a full‐scale training programme. The retention rate at baseline and 37 gestational weeks were met, but the retention rate of 42 days after delivery was not fully achieved. However, there is an imbalance in retention rates between comparison groups. A total of 20 pregnant women (83.33%, 20/24) in the intervention group submitted the questionnaire at 42 days after delivery while 62.5% (15/24) of the pregnant women in the control group completed the questionnaire at 42 days after delivery. Although two text messages were sent to the participants in the control group to remind them to submit the questionnaire, the response rate was still lower than that in the intervention group. One possible reason may be that pregnant women in this group received less attention than participants in the intervention group and therefore were not motivated to return the follow‐up questionnaires. While participants in the intervention group were more inspired to follow the trial procedures because they have been involved in the training after the randomization process.

The adherence rate (66.67%) which was calculated based on the registration of session attendance was lower than anticipated (≥75% to proceed with RCT and ≥62.5% to proceed with changes) which was consistent with the feedback from the semi‐structured interview performed following the intervention with the pregnant women. However, the record in the training diaries showed that 75% (18/24) of pregnant women performed PFMT twice a day at home. This study made efforts to facilitate participants to adhere to the programme and received positive feedback and an adherence rate (75%) with home training. The imbalance of the adherence rate in face‐to‐face sessions and home training may reflect a specific issue related to the face‐to‐face supervision sessions which needs to be further discussed.

### 5.2 Issues needing consideration

#### 5.2.1 Time allocation

The main issue identified by both the midwife and the participants from the interviews was the scheduling of the face‐to‐face sessions. This is the main reason for the low attendance rate in face‐to‐face sessions. In spite of attempts to make it easier for women to attend by scheduling face‐to‐face training on the same day as the antenatal appointment, the participants found it difficult to schedule the time for both attending supervision sessions and doing regular antenatal checks. One possible reason is that pregnant women need to book their next antenatal check with doctors when in receipt of all the results of their regular antenatal checks, consequently they prioritize these instead of attending the training sessions. In addition, time and cost of attending training sessions per se may be a barrier to implementing pelvic floor muscle training for the prevention of UI during pregnancy because the pregnant women may need to take sick leave or leave without pay from work to attend sessions (Brennen et al., 2021). It is believed that if PFMT is not considered as a service priority, it could be difficult for participants to attend additional training due to a lack of time (Salmon et al., 2020). However, having enough time to attend the regular antenatal check was their top priority in the current clinical practice.

The midwife and the pregnant women provided some suggestions about how to improve the attendance rate of the face‐to‐face sessions. One possible way was to provide online training sessions, which would eliminate geographic barriers and increase accessibility (Wu et al., 2022). It was reported that internet‐based PFMT was effective in reducing the number of UI episodes and improving women's health‐specific quality of life in treating stress urinary incontinence (Sjostrom et al., 2013). The improvements in UI symptoms and health‐specific quality of life lasted for 2 years after treatment (Sjostrom et al., 2015). A systematic review which aimed to synthesize the feasibility and effectiveness of PFMT in telerehabilitation found that the severity of UI, pelvic floor muscle strength and quality of life all improved in the pelvic floor telerehabilitation group, in addition, participants were satisfied with telerehabilitation and had good compliance to the intervention (Hao et al., 2024). However, delivering all the training sessions online reduced the opportunities for interactions between group members, which is not consistent with the aim of this study. The participants in this study also mentioned that face‐to‐face sessions need to be retained as they facilitated peer support from the group members and ensured they were monitored and guided by the midwife. Therefore, considering providing some of the sessions online with support from the midwife and integrating the online sessions into the existing programme may have the potential to reduce costs and the time spent in the hospital whilst still maintaining a personalized approach through face‐to‐face sessions and reducing attrition. Whether online sessions will work in maternity settings in China and how to optimize the integration of online sessions will need further exploration. There is a need to go back to the co‐design process based on the findings from this study and for a future definitive trial to include a pilot study with clear stop/proceed criteria to assess the possibility of incorporating face‐to‐face sessions with online sessions.

The other possible approaches suggested by the midwife and the pregnant women were to run loop sessions or provide a booking system for the pregnant women to arrange both the regular antenatal checks and training sessions so that the participants can choose themselves when to attend the sessions. Although the online booking system cannot be changed in the short term, delivering training in a loop may be possible to solve the problem. Mason et al. (2010) tried to provide PFMT sessions at different times and dates for pregnant women who had difficulties with times to choose from, however, the attendance of PFMT sessions was still low (64% of the participants attending only one of the sessions). The authors pointed out that the low attendance of sessions may have been related to the high percentage of pregnant women who worked up until late pregnancy, making it difficult or time‐consuming for pregnant women to attend face‐to‐face sessions (Mason et al., 2010). This may also be a possible factor that has an impact on session attendance in this study. However, this study did not collect information on employment status, so it is not possible to analyse this further.

#### 5.2.2 Fees for the supervised training sessions and increased workload for health professionals

It has been suggested that offering group‐based PFMT during pregnancy to prevent UI after delivery is clinically effective and cost‐effective (Brennen et al., 2021). In addition, to ensure the effectiveness of group intervention, more than two members and less than 15 members in one group were recommended and the optimum number for group intervention is 8–12 members (Ezhumalai et al., 2018). The comparison between group‐based PFMT and individual training for cost‐effectiveness depends on the number of women in a group session and how much the health service charges for the sessions (Brennen et al., 2021). Brennen et al. (2021) found that group‐based PFMT during pregnancy is preferable to individual training for the prevention of UI if at least five women attend the group sessions and the supervised sessions charge $10 per session or at least 13 women attend the group sessions and there is no charge per session. However, effectiveness and cost‐effectiveness should both be considered when deciding the number of women in one group.

Although clinical guidelines recommend undertaking PFMT during pregnancy to prevent UI in late pregnancy and the postpartum period, supervised PFMT instruction in pregnancy is not regularly implemented in practice (Ismail, 2009). For example, supervised PFMT during pregnancy is not available in current practice in Nanjing, China. Although postpartum women have to pay for the training service, the individualized supervision training provided by the hospital was still in short supply due to a shortage of health professionals, resulting in many women who need training supervision after delivery being put on a waiting list. Private postpartum rehabilitation institutions outside the hospital provide PFMT, but the quality of the training cannot be assured and fees vary.

A systematic review which aimed to analyse the most cost‐effective way of providing PFMT to prevent or treat postpartum UI suggested that employment rates of pregnant women in their area of service should be taken into consideration when evaluating the impact of lost productivity costs (Brennen et al., 2021). Training sessions can be provided outside of standard working hours, if possible, to reduce the financial burden of missed work time and childcare. However, providing antenatal PFMT out of standard working hours will additionally increase the workload of already overburdened health professionals. In this study, the midwife suggested that most of the midwives in the hospital did not think it was their responsibility to deliver PFMT to pregnant women as PFMT provision was not included in the antenatal care routine. In addition, physiotherapists played the main roles in providing supervised PFMT to postpartum women in the hospital in China. However, although physiotherapists have traditionally been experts in delivering PFMT, they are no longer frequently involved in the provision of maternity services, pregnant and postpartum women thought midwives were the best professionals to perform antenatal PFMT delivery (Salmon et al., 2020). Visits to the midwife for PFMT supervision have been adopted by other studies and reported to be effective in improving adherence to a PFMT programme (Ahlund et al., 2013; Salmon et al., 2017). The midwife also mentioned that their workload would increase if they tried to provide antenatal pelvic floor muscle training, for example, the number of staffing hours required to deliver intervention sessions (e.g., number of sessions, duration of sessions and number of groups) and assess outcome measures. It was suggested that the national agenda or at least resources at the local level have an enormous effect on the provision of maternity services and as a result, midwives' clinical practice may be guided more by the regulations than by patient need (Salmon et al., 2020). In addition, midwives claimed that the heavy workload prevented them from having the time to apply what they had learned into practice (Filby et al., 2016).

### 5.3 Sample size calculation

Sample size calculation based on ICIQ UI‐SF scores from this study has determined that 49 women would be needed in each arm of a future definitive trial to detect a mean difference in ICIQ UI‐SF scores (a minimum clinically important difference of 2.52) (Lim et al., 2019) at 5‐month post‐randomization (42 days after delivery) with a two‐sided significance level of 5% and a power of 90% with 1:1 allocation. A minimum of 62 pregnant women should be included in each arm in order to account for a drop‐out rate of roughly 27.08% at 42 days after delivery.

### 5.4 The strength of PFMT

Peer support between the group members was mentioned by the participants many times in the interview. Delivering training in groups can foster peer support and the discussion between the participants can increase the motivation of doing exercise and reduce the feeling of isolation (de Oliveira Camargo et al., 2009). In addition, preliminary efficacy analysis based on the small sample size showed that group‐based pelvic floor muscle training had a protective effect in decreasing the impact of UI at 42 days after delivery. However, whether the group‐based PFMT is more effective than individualized training or at least not inferior to individualized training needs to be assessed through future studies.

### 5.5 Limitations of this study

This feasibility study provided mainly descriptive preliminary findings which should be assessed in a large‐scale effectiveness randomized controlled trial. This study included a small sample size and recruited nulliparous women from one hospital in Nanjing City. The education level of the sample showed a skew in that most of the pregnant women (47/48) had received higher education so the results cannot be generalized to the wider population of pregnant women in China. The principal researcher, who also performed the interview and analysed the data of the interview, purposively sampled participants for the interview, which may bring bias in analysing the acceptability of the intervention.

### 5.6 Recommendations for future research

This study was grounded in evidence and involved stakeholders throughout the development process of the group‐based PFMT programme. The next step of the study would be to refine the programme according to the findings of the feasibility study. The refinements include the revision of education on PFMT, improvements to the slides used in the training sessions, a change in the collection method of the outcome measure and considering integrating online training sessions with face‐to‐face training sessions. Findings from this study imply that different combinations of online sessions and face‐to‐face sessions may be required. For example, provide two face‐to‐face sessions, which can be scheduled by pregnant women themselves and two online sessions or keep four face‐to‐face sessions for skill training but to provide online education on PFMT. The attempt and adaptation in the delivery mode of sessions can potentially improve the attendance rate of training sessions, thus leading to an improvement in women's adherence to the training programme. One of the limitations of this study was that the participants were recruited from one hospital and mainly had university degree or above. Future research should consider recruiting participants from multiple backgrounds and considering their views on group‐based PFMT to make the programme relevant for a broader group of pregnant women and provide a greater understanding of the group‐based PFMT programme.

### 5.7 Implication for policy and practice

This study demonstrated that engagement in a PFMT programme can be a challenge for midwives in current clinical practice. It is important to ensure additional time is available for midwives to reflect on their work and improve their skills, although it is recognized that midwifery services are often understaffed. Therefore, support and training should be provided to health professionals to enable them to have the access to programme development workshops, thus leading to an improvement in their research skills and capabilities to keep pace with current requirements.

## 6 CONCLUSION

This is the first study to assess the feasibility of group‐based pelvic floor muscle training in pregnant women in China. This study shows that (i) it is feasible to deliver group‐based pelvic floor muscle training to pregnant women in China, (ii) pregnant women find group‐based pelvic floor muscle training acceptable and (iii) pregnant women may benefit from this intervention, which has been identified as effective in other populations. An adequately powered randomized controlled trial is needed to assess the clinical and cost‐effectiveness of group‐based pelvic floor muscle training in pregnant women in China versus standard care. To overcome the barriers to implementation identified by the midwife and participants and assess whether the group‐based pelvic floor muscle training programme can be used in clinical practice, refinements need to be made to the programme.

## 7 FUNDING INFORMATION

This study did not receive any funding.

## CONFLICT OF INTEREST STATEMENT

The authors have no conflict of interest related to this publication.

### PEER REVIEW

The peer review history for this article is available at https://www.webofscience.com/api/gateway/wos/peer‐review/10.1111/jan.16365.

## Supporting information


Table S1.

Table S2.

Table S3.

## Data Availability

The data that support the findings of this study are available on request from the corresponding author. The data are not publicly available due to privacy or ethical restrictions.

## References

[jan16365-bib-0001] Ahlund, S. , Nordgren, B. , Wilander, E. L. , Wiklund, I. , & Friden, C. (2013). Is home‐based pelvic floor muscle training effective in treatment of urinary incontinence after birth in primiparous women? A randomized controlled trial. Acta Obstetricia et Gynecologica Scandinavica, 92, 909–915. 23672520 10.1111/aogs.12173

[jan16365-bib-0002] Aoki, Y. , Brown, H. W. , Brubaker, L. , Cornu, J. N. , Daly, J. O. , & Cartwright, R. (2017). Urinary incontinence in women. Nature Reviews Disease Primers, 3, 17097. 10.1038/nrdp.2017.97 29143807

[jan16365-bib-0003] Biswas, B. , Bhattacharyya, A. , Dasgupta, A. , Karmakar, A. , Mallick, N. , & Sembiah, S. (2017). Urinary incontinence, its risk factors, and quality of life: A Study among women aged 50 years and above in a rural health Facility of West Bengal. Journal of Midlife Health, 8, 130–136. 28983160 10.4103/jmh.JMH_62_17 PMC5625577

[jan16365-bib-0004] Bland, D. R. , Dugan, E. , Cohen, S. J. , Preisser, J. , Davis, C. C. , Mcgann, P. E. , Suggs, P. K. , & Pearce, K. F. (2003). The effects of implementation of the Agency for Health Care Policy and Research Urinary incontinence guidelines in primary care practices. Journal of the American Geriatrics Society, 51, 979–984. 12834518 10.1046/j.1365-2389.2003.51311.x

[jan16365-bib-0005] Braun, V. , & Clarke, V. (2006). Using thematic analysis in psychology. Qualitative Research in Psychology, 3, 77–101.

[jan16365-bib-0006] Brazier, J. , Czoski‐Murray, C. , Roberts, J. , Brown, M. , Symonds, T. , & Kelleher, C. (2008). Estimation of a preference‐based index from a condition‐specific measure: The King's health questionnaire. Medical Decision Making, 28, 113–126. 17641139 10.1177/0272989X07301820

[jan16365-bib-0007] Brennen, R. , Frawley, H. C. , Martin, J. , & Haines, T. P. (2021). Group‐based pelvic floor muscle training for all women during pregnancy is more cost‐effective than postnatal training for women with urinary incontinence: Cost‐effectiveness analysis of a systematic review. Journal of Physiotherapy, 67, 105–114. 33771484 10.1016/j.jphys.2021.03.001

[jan16365-bib-0008] Cacciari, L. P. , Kouakou, C. R. , Poder, T. G. , Vale, L. , Morin, M. , Mayrand, M. H. , Tousignant, M. , & Dumoulin, C. (2022). Group‐based pelvic floor muscle training is a more cost‐effective approach to treat urinary incontinence in older women: Economic analysis of a randomised trial. Journal of Physiotherapy, 68, 191–196. 35753969 10.1016/j.jphys.2022.06.001

[jan16365-bib-0009] D'ancona, C. , Haylen, B. , Oelke, M. , Abranches‐Monteiro, L. , Arnold, E. , Goldman, H. , Hamid, R. , Homma, Y. , Marcelissen, T. , Rademakers, K. , Schizas, A. , Singla, A. , Soto, I. , Tse, V. , de Wachter, S. , Herschorn, S. , & Standardisation Steering Committee, I. C. S., The, I. C. S. W. G. O. T. F. M. L. U. T., Pelvic Floor, S. & Dysfunction . (2019). The international continence society (ICS) report on the terminology for adult male lower urinary tract and pelvic floor symptoms and dysfunction. Neurourology and Urodynamics, 38, 433–477. 30681183 10.1002/nau.23897

[jan16365-bib-0010] de Oliveira Camargo, F. , Rodrigues, A. M. , Arruda, R. M. , Ferreira Sartori, M. G. , Girao, M. J. , & Castro, R. A. (2009). Pelvic floor muscle training in female stress urinary incontinence: Comparison between group training and individual treatment using PERFECT assessment scheme. International Urogynecology Journal and Pelvic Floor Dysfunction, 20, 1455–1462. 19690792 10.1007/s00192-009-0971-1

[jan16365-bib-0011] Dumoulin, C. , Morin, M. , Danieli, C. , Cacciari, L. , Mayrand, M. H. , Tousignant, M. , Abrahamowicz, M. , Urinary, I. , & Aging Study, G . (2020). Group‐based vs individual Pelvic Floor muscle training to treat Urinary incontinence in older women: A randomized clinical trial. JAMA Internal Medicine, 180, 1284–1293. 32744599 10.1001/jamainternmed.2020.2993 PMC7400216

[jan16365-bib-0012] Ezhumalai, S. , Muralidhar, D. , Dhanasekarapandian, R. , & Nikketha, B. S. (2018). Group interventions. Indian Journal of Psychiatry, 60, S514–S521. 29540924 10.4103/psychiatry.IndianJPsychiatry_42_18 PMC5844165

[jan16365-bib-0013] Filby, A. , Mcconville, F. , & Portela, A. (2016). What prevents quality midwifery care? A systematic mapping of barriers in low and middle income countries from the provider perspective. PLoS One, 11, e0153391. 27135248 10.1371/journal.pone.0153391 PMC4852911

[jan16365-bib-0014] Frechette‐Chaine, E. , Mercier, J. , Fraser, S. , Southall, K. , Morin, M. , & Dumoulin, C. (2018). Psychosocial factors influencing physiotherapeutic adherence to group‐based or individualized pelvic floor rehabilitation: Perceptions of older women with urinary incontinence. Neurourology and Urodynamics, 37, S273–S274.

[jan16365-bib-0015] Hao, J. , Yao, Z. , Remis, A. , Huang, B. , Li, Y. , & Yu, X. (2024). Pelvic floor muscle training in telerehabilitation: A systematic review and meta‐analysis. Archives of Gynecology and Obstetrics, 309, 1753–1764. 38340157 10.1007/s00404-024-07380-x

[jan16365-bib-0016] Huang, Q. , Cao, J. , Liu, L. , & Ye, L. (2016). The prevalence and associated risk factors of urinary incontinence in pregnant and postnatal women in Wuhan city. Maternal and Child Health Care of China, 31, 598–599.

[jan16365-bib-0017] Ismail, S. I. (2009). An audit of NICE guidelines on antenatal pelvic floor exercises. International Urogynecology Journal and Pelvic Floor Dysfunction, 20, 1417–1422. 19669683 10.1007/s00192-009-0967-x

[jan16365-bib-0018] Lim, R. , Liong, M. L. , Lim, K. K. , Leong, W. S. , & Yuen, K. H. (2019). The minimum clinically important difference of the international consultation on incontinence questionnaires (ICIQ‐UI SF and ICIQ‐LUTSqol). Urology, 133, 91–95. 31415780 10.1016/j.urology.2019.08.004

[jan16365-bib-0019] Mason, L. , Roe, B. , Wong, H. , Davies, J. , & Bamber, J. (2010). The role of antenatal pelvic floor muscle exercises in prevention of postpartum stress incontinence: A randomised controlled trial. Journal of Clinical Nursing, 19, 2777–2786. 20846227 10.1111/j.1365-2702.2010.03297.x

[jan16365-bib-0020] Moossdorff‐Steinhauser, H. F. A. , Berghmans, B. C. M. , Spaanderman, M. E. A. , & Bols, E. M. J. (2021). Prevalence, incidence and bothersomeness of urinary incontinence in pregnancy: A systematic review and meta‐analysis. International Urogynecology Journal, 32, 1633–1652. 33439277 10.1007/s00192-020-04636-3 PMC8295103

[jan16365-bib-0021] Nice . (2019). NICE guidance‐Urinary incontinence and pelvic organ prolapse in women: Management: (c) NICE (2019) Urinary incontinence and pelvic organ prolapse in women: Management. BJU International, 123, 777–803. 31008559 10.1111/bju.14763

[jan16365-bib-0022] Salmon, V. E. , Hay‐Smith, E. J. , Jarvie, R. , Dean, S. , Oborn, E. , Bayliss, S. E. , Bick, D. , Davenport, C. , Ismail, K. M. , Macarthur, C. , Pearson, M. , & Study, A. (2017). Opportunities, challenges and concerns for the implementation and uptake of pelvic floor muscle assessment and exercises during the childbearing years: Protocol for a critical interpretive synthesis. Systematic Reviews, 6, 18. 28122608 10.1186/s13643-017-0420-z PMC5267404

[jan16365-bib-0023] Salmon, V. E. , Hay‐Smith, E. J. C. , Jarvie, R. , Dean, S. , Terry, R. , Frawley, H. , Oborn, E. , Bayliss, S. E. , Bick, D. , Davenport, C. , Macarthur, C. , & Pearson, M. (2020). Implementing pelvic floor muscle training in women's childbearing years: A critical interpretive synthesis of individual, professional, and service issues. Neurourology and Urodynamics, 39, 863–870. 31845393 10.1002/nau.24256 PMC7079154

[jan16365-bib-0024] Sjostrom, M. , Umefjord, G. , Stenlund, H. , Carlbring, P. , Andersson, G. , & Samuelsson, E. (2013). Internet‐based treatment of stress urinary incontinence: A randomised controlled study with focus on pelvic floor muscle training. BJU International, 112, 362–372. 23350826 10.1111/j.1464-410X.2012.11713.x PMC3798106

[jan16365-bib-0025] Sjostrom, M. , Umefjord, G. , Stenlund, H. , Carlbring, P. , Andersson, G. , & Samuelsson, E. (2015). Internet‐based treatment of stress urinary incontinence: 1‐ and 2‐year results of a randomized controlled trial with a focus on pelvic floor muscle training. BJU International, 116, 955–964. 25683075 10.1111/bju.13091 PMC4690161

[jan16365-bib-0026] Skivington, K. , Matthews, L. , Simpson, S. A. , Craig, P. , Baird, J. , Blazeby, J. M. , Boyd, K. A. , Craig, N. , French, D. P. , Mcintosh, E. , Petticrew, M. , Rycroft‐Malone, J. , White, M. , & Moore, L. (2021). A new framework for developing and evaluating complex interventions: Update of Medical Research Council guidance. BMJ, 374, n2061. 34593508 10.1136/bmj.n2061 PMC8482308

[jan16365-bib-0027] Wang, Y. (2009). The prevalence of UI and associated factors towards UI in pregnant women and postpartum women.

[jan16365-bib-0028] Wu, J. , Rajesh, A. , Huang, Y. N. , Chhugani, K. , Acharya, R. , Peng, K. , Johnson, R. D. , Fiscutean, A. , Robles‐Espinoza, C. D. , de la Vega, F. M. , Bao, R. , & Mangul, S. (2022). Virtual meetings promise to eliminate geographical and administrative barriers and increase accessibility, diversity and inclusivity. Nature Biotechnology, 40, 133–137. 10.1038/s41587-021-01176-z 34966181

[jan16365-bib-0029] Yang, X. , Zhang, A. , Zhu, R. , Sayer, L. , Bassett, S. , & Woodward, S. (2023). Group‐based PFMT programme for preventing and/or treating UI in pregnant women: Protocol of a randomized controlled feasibility study. Pilot and Feasibility Studies, 9, 180. 37907990 10.1186/s40814-023-01410-2 PMC10617193

[jan16365-bib-0030] Zengin, N. (2010). Urinary incontinence prevalence and risk factors in women. Firat Saglik Hizmetleri Dergisi, 5, 45–60.

